# Supplementary material for: A study on turbulence characteristics of a rectangular three-dimensional wall jet in a confined space using particle image velocimetry
Source: PLoS One. 2026 May 8;21(5):e0348138. doi: 10.1371/journal.pone.0348138 (PMC13155588; doi:10.1371/journal.pone.0348138)
Supplement: S1 File — (DOCX) [file pone.0348138.s001.docx]

**Nomenclature**

*λ_l_*  horizontal scale

*λ_h_*  vertical scale

*λ_u_* velocity scale

*d* square root of the jet exit area

*U*_0_  jet exit velocity

Re Reynolds number based on *U*_0_ and *d*

*ν* kinetic viscosity of water

*H* submerged depth

*x* streamwise direction in the coordinate system

*y* wall-normal direction in the coordinate system

*z*  spanwise direction in the coordinate system

*U*  streamwise mean velocity

*V*  wall-normal mean velocity

*W*  spanwise mean velocity

*u* streamwise turbulence intensity

*v* wall-normal turbulence intensity

*w* spanwise turbulence intensity

*uv*  Reynolds shear stress

*U_m_*  local maximum streamwise mean velocity

*y_m_* wall-normal location where *U_m_* occurs

*y*_0.5_ wall-normal location where 0.5*U_m_* occurs

*z_0.5_* spanwise location where 0.5*U_m_* occurs
