# Supplementary material for: A study on turbulence characteristics of a rectangular three-dimensional wall jet in a confined space using particle image velocimetry
Source: PLoS One. 2026 May 8;21(5):e0348138. doi: 10.1371/journal.pone.0348138 (PMC13155588; doi:10.1371/journal.pone.0348138)
Supplement: S1 Table — (DOCX) [file pone.0348138.s002.docx]

**S1 Table. Partial** **statistical data of** **side orifices longitudinal culvert filling and emptying systems**

| **Sl. no.** | **Names of ship locks** | **Scales of chamber**  **(long × wide)**  **(m)** | **Initial depth**  **(m)** | **Designing head**  **(m)** | **Filling time**  **(min)** |
| --- | --- | --- | --- | --- | --- |
| **1** | Wheeler | 122×18.3 | 3.96 | 16.4 | 12.0 |
| **2** | Vallee | 204×33.5 | 3.96 | 6.7 | 9.9 |
| **3** | Eisen Howen | 244×24.4 | 9.45 | 15.7 | 8.4 |
| **4** | Jackson | 204×33.5 | 3.96 | 10.86 | 10.7 |
| **5** | Colombia | 154×25 | 4.27 | 7.65 | 9.8 |
| **6** | First-line of Guiping | 186×23 | 3.5 | 9.96 | 8.0 |
| **7** | Shaying | 130×12 | 2.5 | 12.0 | 8.50 |
| **8** | Naji | 190×12 | 3.5 | 13.91 | 8.0 |
| **9** | First-line of Dayuandu | 180×23 | 3.0 | 11.2 | 8.7 |
| **10** | First-line of Guigang | 190×23 | 3.5 | 14.5 | 8.3 |
| **11** | Tongnan | 120×12 | 2.5 | 7.0 | 8.0 |
