# Supplementary material for: A study on turbulence characteristics of a rectangular three-dimensional wall jet in a confined space using particle image velocimetry
Source: PLoS One. 2026 May 8;21(5):e0348138. doi: 10.1371/journal.pone.0348138 (PMC13155588; doi:10.1371/journal.pone.0348138)
Supplement: Attachment — Submitted filename: Response to Reviewers.docx [file pone.0348138.s004.docx]

**Authors' detailed response to the reviewers**

**(July 2025)**

**General reply to the Editor**

We sincerely appreciate (the Editor) and the reviewers for the comprehensive review of our research paper. First of all, we offer our most sincere apologies to you. Due to a large number of materials need to prepare for undergraduate professional evaluation recently, there has been a delay in submitting the revised manuscript. We seriously considered all the comments given by the reviewers and you (the Editor) for revising the paper to rectify the shortages and enhance the quality. All the comments are interesting and helped to improve the content and quality of the paper. According to the comments of the editor and reviewers, we have revised the paper draft both grammatically and technically. We have given a detailed response to the reviewers' comments. What is more, we have stated the financial disclosure of the Postdoctoral Science Foundation of China in the cover letter. If there is any more comment, please suggest them, and we will be delighted to make further improvements to our paper.

**General reply to the reviewers:**

Thank you very much for your further valuable suggestions on our paper draft. Your comments are very helpful for us in further improving the paper's quality, and we sincerely appreciate your time and effort spent inspecting our manuscript. We have carefully revised our manuscript according to your comments in this revision. The detailed responses to each comment are listed below in the “***Specific reply to the Reviewers’ comments one by one***”. To facilitate your review, we have additionally attached a draft with all the correction marks in the submission package. We genuinely hope our paper can contribute to the understanding of turbulence characteristics of a three-dimensional confined wall jet.

**Specific reply to the Reviewers’ comments one by one**

**Reviewer#1:**

**(The specific Reviewer Number may be different between the author side and the reviewer side)**

**Reviewer#1's comment No.1:** How this paper is different than the published two papers (ref. 20 and 21) by the authors (except the Reynolds stresses)?

**Reply:** We greatly appreciate your valuable comment. The differences between the published two papers (ref.20 and 21) and this paper are extracted below:

The mean flow characteristics of a three-dimensional (3D) confined wall jet issuing from a rectangular orifice were mainly studied in the published two papers (ref.20 and 21). The results of the published two papers showed that the mean flow development of a confined jet remained unaffected by the vertical wall until *x/d*=16, while the other regions were significantly characterized by the vertical wall. Therefore, mean flow characteristics in region Ⅱ and region III were presented emphatically in the above published papers, including the definition of three regions along the streamwise direction, profiles of mean velocities, variations of velocity-half-height and velocity-half-width and decay of the maximum velocity. The influence of Reynolds numbers on mean characteristics in region Ⅱ and region III was discussed in Ref. [20], while the influence of submerged depths in region Ⅱ was studied significantly in Ref. [21]. It is noted that mean characteristics in region Ⅱ reported by Ref. [20] and [21] were similar to those of the radial decay region. Therefore, the results showed that the vertical wall helped the 3D wall jet to develop. According to our previous studies, the influence mechanism of the vertical wall for mean flow development was set up, which is the research foundation of this paper.

In general, fluctuating motion is vital for understanding shear layer formation, interaction between the inner and outer layers, and energy dissipation. However, turbulence characteristics of the confined jet affected by the vertical wall are still not clear, which are not reported in the published two papers. To explore this problem, profiles of turbulence intensities, profiles of Reynolds stresses, the influence of Reynolds numbers and submerged depths on turbulence characteristics is needed to investigate urgently in region Ⅱ and region III. Based on mean flow characteristics studied in the published two papers, this study focuses on showing turbulence characteristics with different Reynolds numbers and submerged depths in region Ⅱ and region III.

To facilitate your further review, we have made the following modifications in the introduction section:

*Especially, Ref. [20] and [21] focused on showing mean characteristics with different Reynolds numbers and submerged depths in the regions affected by the vertical wall. These studies showed that the mean flow development of a confined jet remained unaffected by the vertical wall until x/d=16, while the other regions were significantly characterized by the vertical wall. Fig 2* *shows flow regions of a 3D wall jet in a semi-infinite and confined space. Mean flow characteristics in region Ⅱ and region III were presented emphatically, including the definition of three regions along the streamwise direction, profiles of mean velocities, variations of velocity-half-height and velocity-half-width and decay of the maximum velocity. The influence of Reynolds numbers on mean characteristics in region Ⅱ and region III was discussed in Ref. [20], while the influence of submerged depths in region Ⅱ was studied significantly in Ref. [21]. It is noted that mean characteristics in region Ⅱ reported by Ref. [20] and [21] were similar to those of the radial decay region. Therefore, the results showed that the vertical wall helped the 3D wall jet to develop. According to previous investigations, the influence mechanism of the vertical wall for mean flow developments was set up, which is the research foundation for studying turbulence characteristics of a 3D confined wall jet.*

(a)

(b)

**Fig 2. A sketch of flow regions of a 3D wall jet in a semi-infinite and confined space, (a) a semi-infinite space (after Sforza and Herbst [9]) and (b) a confined space (after Zhang et al. [21]).**

We sincerely appreciate your comment. Please check the mentioned parts for more details. If there is any more comment, please inform us, and we will be very glad to make further progress.

**Reviewer#1's comment No.2:** It is not clear as how the test tank is supplied the water from the top tank. Neither the location and position of constant head skimming weir are discussed nor is the flow rates. It is very difficult to understand the schematic diagram of the setup. Moreover, there is no discussion on the effect of flow rates on the velocity profiles, as this is very important while studying the turbulent characteristics of a wall jet.

**Reply:** We greatly appreciate your insightful feedback. Following your feedback, we have redrawn the schematic diagram of the experimental setup, as shown in Fig 3. Firstly, in general, external water was pumped to the top tank, whose depth was measured by a water level probe when filling. To remain a constant water level of the top tank, excess water returned to the bottom tank through the overflow plate. The supply-return water paths were shown in Fig 3. Note that the aim of the supply-return facilities was to provide a stable incident flow rate for the test tank. Secondly, as shown in Fig 3, the initial positions of four constant-head skimming weirs were located at a height of 300 mm above the top of the test tank. At the same time, the flexible pipes connected constant-head skimming weirs to the bottom tank, creating a path for overflow. Through the rise and fall of constant-head skimming weirs, experimental conditions for different water levels in the test section were achieved. What is more, the flow rates were discussed in the section “*Test conditions and test cases*”. The flow rates were tested by an electromagnetic flowmeter to obtain different Reynolds numbers of the jet exit, which were 0.0891L/s, 0.1091 L/s, and 0.1311 L/s, respectively. Finally, we strongly agree with your professional comment that the effect of flow rates on the velocity profiles is very important while studying the turbulent characteristics of a wall jet. In response to your feedback, we provide a detail explanation. It is showed that the effect of flow rates on the velocity profiles is very important while studying the turbulent characteristics of a 3D wall jet. The effect of flow rates on the velocity profiles with different Reynolds numbers had been discussed detailly in previous studies (Ref. [20] and [21]). In particular, the streamwise velocity profiles were well collapsed and consistent with Gaussian distribution in region Ⅱ, which were independent of Reynolds numbers and submerged depths. However, after the wall jet entered region III, the similarity of U profiles was disappeared owing to the influence of the vertical wall. As mentioned above, this study did not show the effect of flow rates on the velocity profiles.

To facilitate your further review, we have made the following modifications in the paper:

(1) *External water was pumped to the top tank, whose depth was measured by a water level probe when filling. To remain a constant water level of the top tank, excess water returned to the bottom tank through the overflow plate. The supply-return water paths were shown in Fig 3. Note that the aim of the supply-return facilities was to provide a stable incident flow rate for the test tank.*
(2) *The initial positions of four constant-head skimming weirs were located at a height of 300 mm above the top of the test tank (Fig 3). At the same time, the flexible pipes connected constant-head skimming weirs to the bottom tank, creating a path for overflow. Through the rise and fall of constant-head skimming weirs, experimental conditions for different water levels in the test section were achieved.*

**Fig 3. A sketch of the confined wall jet facilities (NB: All dimensions in mm).**

(3) *It is showed that the effect of flow rates on the velocity profiles is very important while studying the turbulent characteristics of a 3D wall jet. The effect of flow rates on the velocity profiles with different Reynolds numbers had been discussed detailly in previous studies (Ref. [20] and [21]). In particular, the streamwise velocity profiles were well collapsed and consistent with Gaussian distribution in region Ⅱ, which were independent of Reynolds numbers and submerged depths. However, after the wall jet entered region III, the similarity of U profiles was disappeared owing to the influence of the vertical wall.*

To better understand the schematic diagram of the setup, we have made a diagram that shows the setting processes of the flow rate and water level, as shown in Fig 4.

**Fig 4. The setting processes of the flow rate and water level for each test case**

We sincerely appreciate your comment. Please check the mentioned parts for more details. If there is any more comment, please inform us, and we will be very glad to make further progress.

**Reviewer#1's comment No.3:** A valve is also shown in the diagram near the exit of the orifice; this affects the turbulent characteristics drastically. But, there is no discussion on the turbulent fluctuation at the exit of the orifice.

**Reply:** We greatly appreciate your insightful feedback. Your comment greatly contributes to the strictness of this paper. In fact, a value of the long rectangular pipe plays the most important role for controlling the flow rate. The valve near the exit of the orifice is opened fully in the experiments, which is useless. Following your feedback, to promote the strictness of this study, we have removed this valve near the exit of the orifice in Fig 3.

We sincerely appreciate your comment. Please check the mentioned parts for more details. If there is any more comment, please inform us, and we will be very glad to make further progress.

**Reviewer#1's comment No.4:** How the near wall and outer region of the wall are selected as mentioned in lines 84-90? A detailed discussion on this is required.

**Reply:** We greatly appreciate your insightful feedback. Following your feedback, we provide a detail discussion. Firstly, during the filling of side orifices longitudinal culvert filling and emptying systems frequently employed in ship locks, the upstream flow with a certain initial momentum through side orifices injects into the chamber, resulting in the production of side orifices jets. A confined space is formed because of two lock walls which are defined as vertical walls in this study, based on the design manual of ship locks made by U.S. Army Corps of Engineers. The test section is the core component of confined wall jet facilities, which recreates a confined 3D wall jet as accurately as feasible when water filling in lock chambers. Therefore, the design of the width with the test section which must simulate exactly the confined space is particularly important. A summary of typical ship locks with side orifices longitudinal culvert filling and emptying systems is provided in Table 1 [8]. It is showed that most real widths of lock chambers are within the range of 12m-25m, resulting in the fact that 20m is selected as the prototype size to build the width of the test section in this study. Secondly, considering the experimental field, range of laser irradiation, observation range of the CCD camera, accuracy requirements for images, etc., the test section was designed according to the normal model that should obey the similarity law of gravity, and its geometric scale was determined to be 1:50. i.e., *λ_l_*=*λ_h_*=50, *λ_u_*=*λ_l_*^1/2^=7.07. Note that *λ_l_* represents the horizontal scale, *λ_h_* represents the vertical scale, and *λ_u_* represents the velocity scale, respectively. Its width was determined to be 400mm by calculation. Finally, due to difficulties in reconstructing the whole length of the lock chamber, the length of the test section was designed to be twice the width to facilitate better diffusion of a 3D wall jet in the spanwise direction. Moreover, the test section was set as 300mm high to provide greater activity space for constant-head skimming weirs. The side orifice was rectangular in shape with 14 mm wide as well as 16 mm high.

**Table 1.** Typical ship locks with side orifices longitudinal culvert filling and emptying systems (after Zhang et al. [8])

| **Serial number** | **Names of ship locks** | **Scales of chambers**  **（Length and Width）（m）** | **Initial depths（m）** | **Designed heads（m）** | **Filling time（min）** |
| --- | --- | --- | --- | --- | --- |
| 1 | Vallee | 204×33.5 | 3.96 | 6.7 | 9.9 |
| 2 | Jackson | 204×33.5 | 3.96 | 10.86 | 10.7 |
| 3 | Colombia | 154×25 | 4.27 | 7.65 | 9.8 |
| 4 | Eisen Howen | 244×24.4 | 9.45 | 15.7 | 8.4 |
| 5 | First line of Guiping | 186×23 | 3.5 | 9.96 | 8.0 |
| 6 | First line of Dayuandu | 180×23 | 3.0 | 11.2 | 8.7 |
| 7 | First line of Guigang | 190×23 | 3.5 | 14.5 | 8.3 |
| 8 | Wheeler | 122×18.3 | 3.96 | 16.4 | 12.0 |
| 9 | Naji | 190×12 | 3.5 | 13.91 | 8.0 |

To facilitate your further review, we have made the following modifications in the paper:

*A confined jet space is formed because of two lock walls which are defined as vertical walls in this study, based on the design manual of ship locks made by U.S. Army Corps of Engineers. The test section is the core component of confined wall jet facilities, which recreates a confined 3D wall jet as accurately as feasible when water filling in lock chambers. Therefore, the design of the width with the test section which must simulate exactly the confined space is particularly important. A summary of typical ship locks with side orifices longitudinal culvert filling and emptying systems is provided in Table 1 [8]. It is showed that most real widths of lock chambers are within the range of 12m-25m, resulting* *in the fact that 20m is selected as the prototype size to build the width of the test section in this study.* *Considering the experimental field, range of laser irradiation, observation range of the CCD camera, accuracy requirements for images, etc., the test section was designed according to the normal model which should obey the similarity law of gravity, and its geometric scale was determined to be 1:50.* i.e., *λ_l_=λ_h_=50, λ_u_=λ_l_^1/2^=7.07. Note that λ_l_ represents the horizontal scale, λ_h_ represents the vertical scale, and λ_u_ represents the velocity scale, respectively. Its width was determined to be 400mm by calculation. Due to difficulties in reconstructing the whole length of the lock chamber, the length of the test section was designed to be twice the width to facilitate better diffusion of a 3D wall jet in the spanwise direction. Moreover, the test section was set as 300mm high to provide greater activity space for constant-head skimming weirs. The side orifice was rectangular in shape with 14 mm wide as well as 16 mm high.*

We sincerely appreciate your comment. Please check the mentioned parts for more details. If there is any more comment, please inform us, and we will be very glad to make further progress.

**Reviewer#1's comment No.5:** In most of the figures, the authors talk about the collapsing of profiles within a short range of x/d (like x/d = 21 and 22, x/d = 24 and 25 and so), which is of no meaning. The authors must refer to the papers published on the self-similarity and notice that within such a small range we cannot talk about the self-similarity (usually we present the result with a distance of at least x/d = 5).

**Reply:** We greatly appreciate your insightful feedback. Your comment greatly contributes to the strictness and quality of this paper. Here, we provide a detail explanation. Firstly, we strongly agree with your professional viewpoint that the self-similarity was usually presented with a distance of at least *x/d* = 5. According to previous studies of turbulence characteristics for a 3D wall jet in a semi-infinite space (like Ref. [22-27]), it is showed that profiles of turbulence intensities and Reynolds shear stresses in both the symmetry and lateral planes were well collapsed in the radial decay (RD) region. Based on our studies (Ref. [20-21]), the vertical wall helped the 3D wall jet to develop and mean flow characteristics in region Ⅱ (16≤*x/d*＜24) were similar to the RD region. i.e., Profiles of mean velocity (*U*), variations of velocity-half-height and velocity-half-width and decay of the maximum velocity showed reasonable collapse with a distance *x/d*=8. However, the mean flow developments have to be self-similar before the turbulence quantities refer to earlier findings. The accurate location of the self-similar profile of turbulent characteristics in region Ⅱ is one of the problems that this study aims to solve. Secondly, in the section “Turbulence statistics in region Ⅱ”, taking cases S1 and L1 as an example in Fig 8, it is demonstrated that profiles of turbulence characteristics (19*d*, 20*d*, 21*d*, 22*d* and 23*d*) were well collapsed with a distance *x/d*=5 in both the symmetry and lateral planes. This illustrates that turbulence statistics in region Ⅱ take a longer streamwise distance (3d) to become self-similar than mean characteristics. Thirdly, to explore the effect of Reynolds numbers and submerged depth on turbulence characteristics, we select 21*d* and 22*d* located in 19≤*x/d*＜24 as significant profiles. The results are presented in “Fig 9 Profiles of turbulence intensities and Reynolds shear stresses from case S1, S2, S3, L1, L2, and L3 at selected locations for different Reynolds numbers in the region 19≤*x/d*<24” and “Fig 10 Profiles of turbulence intensities and Reynolds shear stresses from case S3, S6, S9, S12, L3, L6, L9, and L12 at selected locations for different submerged depths in the region 19≤*x/d*<24”. Due to our lack of clearer expression for the self-similarity region, following your feedback, we have uniformly adjusted it to 19≤x/d<24 in the paper. Finally, *x/d* = 24 and 25 located in region Ⅲ were not the self-similarity profiles, but they only have the same trend of change according to our observed results. Following your feedback, a more accurate discussion of the two profiles and other parts in region Ⅲ have been made in the section “Results and discussion” to promote the quality of this study.

(a)	(b)

(c)	(d)

(e)	(f)

**Fig 8. Profiles of turbulence statistics from case S1 and L1 at both in the symmetry and lateral planes within region Ⅱ**

We sincerely appreciate your comment. Please check the mentioned parts for more details. If there is any more comment, please inform us, and we will be very glad to make further progress.

**Reviewer#2:**

**(The specific Reviewer Number may be different between the author side and the reviewer side)**

**Reviewer#2's comment No.1:** How did you ensure that the scaling criteria (1:50 length, 1:7.07 velocity) accurately represented real-world conditions, and what are the potential limitations of applying these results to full-scale systems?

**Reply:** We greatly appreciate your insightful feedback. Following your feedback, we give a detailed explanation. Firstly, A confined jet space is formed because of two lock walls which are defined as vertical walls in this study, based on the design manual of ship locks made by U.S. Army Corps of Engineers. The design objective of the test section is to recreate a confined 3D wall jet as accurately as feasible when water filling in lock chambers. Therefore, the width with the test section which must simulate exactly the confined space is particularly important. Most real widths of lock chambers are within the range of 12m-25m shown in table 1, resulting in the fact that 20m is selected as the prototype size to build the width of the test section in this study. Secondly, considering the experimental field, range of laser irradiation, observation range of the CCD camera, accuracy requirements for images, etc., the test section was designed according to the normal model that should obey the similarity law of gravity, and its geometric scale was determined to be 1:50 in this study. i.e., *λ_l_*=*λ_h_*=50, *λ_u_*=*λ_l_*^1/2^=7.07. Note that *λ_l_* represents the horizontal scale, *λ_h_* represents the vertical scale, and *λ_u_* represents the velocity scale, respectively. Its width was determined to be 400mm by calculation. due to difficulties in reconstructing the whole length of the lock chamber, the length of the test section was designed to be twice the width to facilitate better diffusion of a 3D wall jet in the spanwise direction. Moreover, the test section was set as 300mm high to provide greater activity space for constant-head skimming weirs. In summary, the width of the test section may represent most real chamber widths, which is able to restore significantly the side orifice jet in the lock chambers when filling and ensure the correctness of the jet experiments in this study. In fact, when conducting an intensive study on transient motion and vortex structure of the 3D confined wall jet in the future, the scale will be further adjusted according to the requirements of research accuracy in different regions. Finally, there are two main potential limitations to apply these results to full-scale systems. On the one hand, due to the length of the test section has not been fully restored to the length of lock chambers, to a certain extent, the expansion range of the jet in the lateral plane may be larger, that will mix more surrounding stationary fluid to promote turbulence level. On the other hand, in full-scale systems, irregular maintenance channels, floating mooring columns, and structural joints, etc., located at the lock walls may affect the flow structure of the jet in the symmetry plane.

**Table 1.** Typical ship locks with side orifices longitudinal culvert filling and emptying systems (after Zhang et al. [8])

| **Serial number** | **Names of ship locks** | **Scales of chambers**  **（Length and Width）（m）** | **Initial depths（m）** | **Designed heads（m）** | **Filling time（min）** |
| --- | --- | --- | --- | --- | --- |
| 1 | Vallee | 204×33.5 | 3.96 | 6.7 | 9.9 |
| 2 | Jackson | 204×33.5 | 3.96 | 10.86 | 10.7 |
| 3 | Colombia | 154×25 | 4.27 | 7.65 | 9.8 |
| 4 | Eisen Howen | 244×24.4 | 9.45 | 15.7 | 8.4 |
| 5 | First line of Guiping | 186×23 | 3.5 | 9.96 | 8.0 |
| 6 | First line of Dayuandu | 180×23 | 3.0 | 11.2 | 8.7 |
| 7 | First line of Guigang | 190×23 | 3.5 | 14.5 | 8.3 |
| 8 | Wheeler | 122×18.3 | 3.96 | 16.4 | 12.0 |
| 9 | Naji | 190×12 | 3.5 | 13.91 | 8.0 |

We sincerely appreciate your comment. Please check the mentioned parts for more details. If there is any more comment, please inform us, and we will be very glad to make further progress.

**Reviewer#2's comment No.2:** What were the primary challenges in using PIV for confined wall jet measurements, such as laser reflections or seeding particle dispersion, and how did you mitigate them?

**Reply:** We greatly appreciate your insightful feedback. We agree with your professional comment that laser reflections or seeding particle dispersion are the primary challenges in using PIV for confined wall jet measurements. Firstly, three common methods were used to solve the issue of laser reflections in this study. The first method was to optimize the optical system. An optical filter with a 532nm wavelength matched the laser was applied in front of the CCD camera lens. The aim of the optical filter is to significantly attenuate reflected stray light. Note that the above discussion has been added in this paper. As mentioned in the original manuscript, to limit local dispersion and intensity attenuation of the light sheet, the present PIV experiments were performed in a closed and shaded space of the laboratory. Therefore, the second method was to cover the test area with a black cloth, and prevent stray light from entering the camera. The third method was to apply the PIV algorithm, which has already been mentioned in the study. Specifically, the instantaneous flow field was filtered to help decrease instability throughout the iteration processes and avoid computation result dispersion. In this way, excessively bright pixels were removed from images. Secondly, two methods were applied to address the problem of seeding particle dispersion. The first method was to select reasonable type, size and density of tracer particle. In this study, the flow seeded with 10 μm hollow glass spheres having a density of 1.03 g/mm^3^. On the one hand, according to previous studies (like Ref. [21]), hollow glass spheres with a diameter of 10 μm matched well to the fluid type of water. On the other hand, the density was very close to water, which may avoid settling or floating and maintain good followability during the jet experiments. The second method was to control the particle concentration. In this study, the particle pixel diameter was approximately 2.06 pixels for all the measurements, which was consistent with the value reported in Ref. [28].

We sincerely appreciate your comment. Please check the mentioned parts for more details. If there is any more comment, please inform us, and we will be very glad to make further progress.

**Reviewer#2's comment No.3:** The study shows that turbulence intensities are sensitive to Reynolds numbers and submerged depths in specific regions but not others. Could you provide a detailed explanation of the physical mechanisms responsible for this behaviour?

**Reply:** We greatly appreciate your insightful feedback. Following your feedback, we provide a detailed discussion. Firstly, based on our previous studies, the jet is unaffected by the vertical wall, and it is regarded as a purely 3D wall jet within region Ⅰ. In fact, region Ⅰ includes the potential core region and the characteristic decay region. The jet has not been fully developed in the above two regions. Turbulence characteristics within the two regions had been reported by earlier research (like Ref. [24]). Considerable research focused on turbulence statistics located in the radial decay region. Therefore, it is noted that turbulence statistics of region Ⅱ and region Ⅲ are mainly researched in this study. Secondly, according to our previous studies (Ref. [20-21]), in region Ⅱ, the vertical wall promotes jet development with mean flow characteristics similar to the RD region. Through research results of turbulence characteristics in this study, the region of flow structure similar to the RD region in the 3D confined wall jet is further determined in this study, i.e., 19≤*x/d*<24. Based on considerable earlier experiment research and our results in the study, it demonstrates that turbulence intensities are not sensitive to Reynolds numbers and submerged depths in the RD region, where the jet motion has been fully developed. Finally, in region Ⅲ, the vertical wall significantly confines a 3D wall jet, showing a corner jet and an upward wall jet features when colliding with it. The vertical wall boundary effect which leads to the change of jet shape was the primary physical reason that turbulence intensities were sensitive to Reynolds numbers. A stronger restriction by the vertical wall significantly enhances the turbulence level in region III (24≤*x/d*<27). Specifically, taking 26*d* profile as an example, when the jet gets further closer to the vertical wall, regarding case S3, turbulence intensities at 26*d* increase rapidly. This may be attributed to the change of flow characteristics. i.e., After the jet collides with the vertical wall, most fluids in the symmetry plane will be along the vertical wall to produce an upward wall jet, and the rest will form a backflow. A stronger influence of the backflow and the pressure gradient can may be obtained close to the vertical wall, leading to relatively higher turbulence intensities. Also, regarding case L3, peak values of *u*, *w* at selected locations in the lateral plane within region Ⅲ are much larger than the observation measured at 22*d*. The relatively higher turbulence statistics close to the vertical wall got in the region 24≤*x/d*<27 in comparison with those at 22*d* for case L3 may due to the formation of a corner jet. When the jet collides with the wall, it will proceed along two right-angle sides because of the Coanda effect to produce a corner jet, resulting in the fact in that more fluids are entrained in the spanwise direction, and vortices of various sizes are developed near the vertical wall. This would enhance turbulence quantities in the lateral plane. In addition, a higher Reynolds number causes more ambient fluids mixing around. This would increase turbulence statistics in the lateral plane through a detailed analysis of 24*d* or 25*d* profile from case L1, L2, and L3. Moreover, based on our research, profiles of turbulence intensities both in the symmetry and lateral planes are not affected by small variations in depths within the region Ⅲ. That demonstrates that a small increase in the vertical pressure gradient would not significantly affect the strength of pulsation.

We sincerely appreciate your comment. Please check the mentioned parts for more details. If there is any more comment, please inform us, and we will be very glad to make further progress.

**Reviewer#2's comment No.4:** You observed self-similarity in turbulence statistics in regions 21≤x/d<24, How does this finding compare with self-similarity trends in unconfined wall jets or similar systems?

**Reply:** We greatly appreciate your insightful feedback. Following your feedback, we provide a detailed discussion. In fact, it should be noted that in the region 19≤*x/d*<24, profiles of turbulence statistics show reasonable collapse by a detail analysis of Fig 8 (Profiles of turbulence statistics from case S1 and L1 at both in the symmetry and lateral planes within region Ⅱ). Here, we summary the turbulence features in the region 19≤*x/d*<24. Firstly, profiles of turbulence intensities and Reynolds shear stresses collapse reasonably well in Fig 8. This rule was the same as the unconfined wall jet in the RD region. Secondly, profiles of turbulence statistics are independent of Reynolds numbers and submerged depths as shown in Fig 9 (Profiles of turbulence intensities and Reynolds shear stresses from case S1, S2, S3, L1, L2, and L3 at 21d and 22d for different Reynolds numbers in the region 19≤*x/d*<24) and Fig 10 (Profiles of turbulence intensities and Reynolds shear stresses from case S1, S2, S3, L1, L2, and L3 at 21d and 22d for different submerged depths in the region 19≤*x/d*<24), which are also the same as the unconfined wall jet in the RD region. Finally, the comparison with the previous studies is shown in Fig 11(Profiles of turbulence statistics from case S3 and case L3 at 22d in comparison with previous studies in the RD region). Based on the analysis results in the section “Comparison and discussion with previous studies”, the present data are comparable to the previous studies, and show the same change trend. We also analysed peak values of turbulence quantities in detail. Therefore, the conclusion was made that turbulence characteristics in the region 19≤*x/d*<24 correspond to those in the RD region of the 3D unconfined wall jet according to reviewing these important features of turbulence quantities. Please refer to the section “Comparison and discussion with previous studies”.

We sincerely appreciate your comment. Please check the mentioned parts for more details. If there is any more comment, please inform us, and we will be very glad to make further progress.

**Reviewer#2's comment No.5:** In the region 24≤x/d<27, turbulence profiles show poor collapse. Could secondary flows, wall boundary effects, or other flow interactions explain these deviations?

**Reply:** We greatly appreciate your insightful feedback. Following your feedback, we provide an explanation. As mentioned above, in region Ⅲ, the vertical wall significantly confines a 3D wall jet, showing a corner jet and an upward wall jet features when colliding with it. A stronger restriction by the vertical wall significantly enhances the turbulence level in region III (24≤*x/d*<27). The vertical wall boundary effect which leads to the change of jet shape was also the essential reason that turbulence profiles show poor collapse.

We sincerely appreciate your comment. Please check the mentioned parts for more details. If there is any more comment, please inform us, and we will be very glad to make further progress.

**Reviewer#2's comment No.6:** Based on your findings, what specific design recommendations would you make for improving energy dissipation and minimizing structural wear in confined engineering systems, such as ship locks or dam spillways?

**Reply:** We greatly appreciate your insightful feedback. Following your feedback, based on our findings, we provide some specific design recommendations to improve energy dissipation and minimizing structural wear in ship locks. Specifically, a dissipation baffle similar to the vertical wall boundary is set up at a certain distance from the side orifice. This will promote turbulent developments of the side jet to obtain a higher turbulence level, leading to a faster energy dissipation process. Of course, the distance from the side orifice and quantity of baffles need to be further determined according to actual lock projects, considering the factors such as designed head, filling time and ship lock safe management, etc.

We sincerely appreciate your comment. Please check the mentioned parts for more details. If there is any more comment, please inform us, and we will be very glad to make further progress.

**Reviewer#2's comment No.7:** The study highlights the sensitivity of Reynolds shear stresses to both Reynolds numbers and submerged depths. How can these insights be used to optimize flow management or reduce turbulence-related impacts in confined spaces?

**Reply:** We greatly appreciate your insightful feedback. Following your feedback, we provide some recommendations to optimize flow management in ship locks. Based on our research, peak values of Reynolds stresses show a positive correlation with both Reynolds numbers and submerged depths. In the initial stage of water filling, a larger incident flow rate of the side orifice would be got by quickly opening the water delivery value, leading to higher Reynolds numbers for the jets injected into the lock chamber. Moreover, due to the increase of the flow rate, the depth inside the lock chamber also grows rapidly. This demonstrates that pulsating motion will absorb much more energy from mean motion in this period, resulting in the fact that momentum exchange and transport within the lock chamber are enhanced to improve energy dissipation and ensure the safety of the lock walls, lock gate and mooring ships.

We sincerely appreciate your comment. Please check the mentioned parts for more details. If there is any more comment, please inform us, and we will be very glad to make further progress.

**Reviewer#2's comment No.8:** The reported uncertainties for Reynolds shear stresses near walls (±12.3%) and away from walls (±10.5%) are significant. What steps could be taken to reduce these uncertainties, and how might this improve confidence in your conclusions?

**Reply:** We greatly appreciate your insightful feedback. Following your feedback, we provide some steps to reduce these uncertainties. It should be noted that in the section “PIV system and PIV algorithm” of the manuscript, on the basis of interrogation window sizes and curve fitting algorithm used to calculate the instantaneous vectors and instantaneous images pairs used to calculate the mean velocity and turbulence statistics. Therefore, three steps may be able reduce these uncertainties. The first step is to downsize the interrogation window size. As mentioned in the study, the minimum interrogation window size was 16 pixels×16 pixels, and the horizontal and vertical grid overlap coefficient were set to 0.5 in JFM, so the final resolution was 8 pixels×8 pixels to improve the calculation accuracy. To get more accurate results, the horizontal and vertical grid overlap coefficient were set to 0.4 or 0.3 in JFM, leading to the fact that the final resolution was 6.4 pixels×6.4 pixels or 4.8 pixels×4.8 pixels. Moreover, the number of iterations in the present calculation was changed from 2 to 3. Obviously, the increase of the number of iterations can also effectively reduce uncertainties. The final step was to add the number of instantaneous images. In general, the uncertainties in this study were comparable to those reported in Ref. [28], ensuring the accuracy and availability of the analysis of subsequent turbulence characteristics.

We sincerely appreciate your comment. Please check the mentioned parts for more details. If there is any more comment, please inform us, and we will be very glad to make further progress.

**Reviewer#2's comment No.9:** Have you considered validating your experimental findings with computational fluid dynamics (CFD) simulations? What specific aspects of your study would benefit the most from numerical modelling?

**Reply:** We greatly appreciate your insightful feedback. In fact, computational fluid dynamics (CFD) simulations about turbulent structure of a 3D confined wall jet are important content that we urgently need to carry out in the future. Following your feedback, we provide specific aspects of the present study would benefit the most from numerical modelling. The obvious advantages of numerical simulation technology are that it can build full-scale systems and quickly simulate a large number of experimental conditions for the 3D confined wall jet. Some interesting issues should be conducted systematically on the basis of numerical modelling in the foreseeable future. Firstly, due to the fixed vertical wall boundary in present experiments, the effect of the distance from the vertical wall to the side orifice (*L*) on turbulence characteristics needs to be a detail discussion. A wide range of *L* should be simulated to explore primarily two problems. The one is to study the distributions of turbulence statistics in a stronger confined space by narrowing *L*. The other one is to find the position unaffected by vertical walls by increasing *L*. It is clear that the results would provide specific reference for setting up the dissipation baffle in real ship locks to improve energy dissipation. Secondly, the effect of higher Reynolds numbers and larger variations in depths on turbulence characteristics of the confined jet may be simulated using CFD method, to supplement and enrich research results of this paper. Finally, note that the experimental data in this study is the basis of choosing an appropriate turbulence model when performing numerical modelling. That is also one of the research significances of this paper.

We sincerely appreciate your comment. Please check the mentioned parts for more details. If there is any more comment, please inform us, and we will be very glad to make further progress.

**In general, we sincerely appreciate your recommendations, which have pointed out the direction for our subsequent research. We will continue to improve the quality of this study on the basis of your three recommendations in the future.**

**Reviewer#3:**

**(The specific Reviewer Number may be different between the author side and the reviewer side)**

**Reviewer#3's comment No.1:** Why is the effect of side walls effecting only a few regions. The other regions do not have side walls?

**Reply:** We greatly appreciate your insightful feedback. Following your feedback, we give a detail explanation. Firstly, in early studies of a 3D unconfined wall jet (Ref. [9]), on the basis of decay characteristics of local maximum velocity, the flow regions along the streamwise direction can be divided into the potential core (PC) region, the characteristic decay (CD) region and the radial decay (RD) region, respectively. According to our previous results of mean characteristics for the confined case (Ref. [20-21]), there are also three regions as follows along the streamwise direction.

(1) Region Ⅰ (0≤*x/d*<16): the jet is unaffected by the vertical wall, and it is regarded as a purely 3D wall jet within the region;

(2) Region Ⅱ (16≤*x/d*<24): the vertical wall promotes jet development with mean flow characteristics similar to the RD region;

(3) Region III (24≤*x/d*<27): the vertical wall significantly confines a 3D wall jet, showing a corner jet and an upward wall jet features when colliding with it.

Fig 2 shows flow regions of a 3D wall jet in a semi-infinite and confined space. Therefore, comparing mean characteristics under two cases in detail, it is found that the wall jet flow was characterized by the side wall in the region 16≤*x/d*<24. Therefore, in the present study, turbulence intensities and Reynolds shear stresses are discussed emphatically in the region Ⅱ and region III based on past studies in our laboratory and compared with previous turbulence characteristics in the RD region of the turbulent 3D and 2D wall jet. Moreover, the effects of the vertical wall, Reynolds numbers and the submerged depths on turbulence statistics are is also discussed and reported. Finally, as shown in Fig 2b, the horizontal wall and two vertical walls are the primary boundaries in the jet experiments. Besides, the horizontal wall is similar to the bottom plate of a ship lock (Fig 1), and the vertical wall simulates the lock wall in a ship lock (Fig 1), respectively. To be better understanding, we mark the jet flow direction and the vertical wall in Fig 1. As mentioned above, the other regions no longer have side walls.

**jet flow direction**

**lock wall (vertical wall)**

**lock wall (vertical wall)**

**Fig 1. Schematic diagram of ship locks in operation**

(a)

(b)

**Fig 2. A sketch of flow regions of a 3D wall jet in a semi-infinite and confined space, (a) a semi-infinite space (after Sforza and Herbst [9]) and (b) a confined space (after Zhang et al. [21]).**

We sincerely appreciate your comment. Please check the mentioned parts for more details. If there is any more comment, please inform us, and we will be very glad to make further progress.

**Reviewer#3's comment No.2:** The schematic is not clearly showing the side walls; may be consider highlighting the side walls.

**Reply:** We greatly appreciate your insightful feedback. Following your feedback, we have added the Fig 1(real ship locks) and Fig 2. What is more, we have redrawn the schematic diagram of the experimental setup, as shown in Fig 3. Through Figs 1, 2, 3, the side walls are showed clearly and highlighted.

**Fig 1. Schematic diagram of ship locks in operation**

**Fig 3. A sketch of the confined wall jet facilities (NB: All dimensions in mm).**

We sincerely appreciate your comment. Please check the mentioned parts for more details. If there is any more comment, please inform us, and we will be very glad to make further progress.

**Reviewer#3's comment No.3:** All the results in each of the regions are only presented as a function of y/y1/2 or z/z1/2, it would be nice if spatial evolution of quantities like spread/growth rate etc. are also presented.

**Reply:** We greatly appreciate your insightful feedback. Following your feedback, we provide a detail explanation. As mentioned in original draft, Fig 5 shows a sketch of a 3D confined wall jet and also defines the coordinate system and notations utilized. The coordinate origin (*o*) is located at the center of the bottom of the orifice. *x*, *y* and *z* represent the streamwise, wall-normal and spanwise directions, respectively. *U*, *V*, and *W* represent the mean velocities in each direction, while *u*, *v*, and *w* denote the turbulence intensities. According to Fig 5, *U_m_* is local maximum streamwise mean velocity, *U*_0.5_ is half of *U_m_*, *y_m_* is the wall-normal location where *U_m_* occurs, *z*_0.5_ and *y*_0.5_ are the lateral and wall-normal locations where *U*_0.5_ occurs. Also, note that the *x-o-z* plane at *y*=*y_m_* and *x-o-y* plane at *z=*0 are the lateral plane and symmetry plane, respectively. The *x-o-z* plane at *y*=0 is on the horizontal wall, the *y*-*o*-*z* plane at *x=*0 is the orifice exit, and the *y*-*o*-*z* plane at *x=*400 coincides with the vertical wall. On the basis of the above coordinate system and notations, y/y1/2 and z/z1/2 as abscissa represent the position of observed points on profiles in each region. This representation method is commonly used to research mean and turbulence characteristics in previous jet experiments studies. What is more, spread rates of the 3D confined jet have been studied in our early studies. In Ref. [21], the spread rate of *z*_0.5_ is 0.205, and the spread rate of *y*_0.5_ is 0.043, respectively. Therefore, spread rates of the jet are not presented in this study.

**Fig 5. Schematic diagram of a 3D confined wall jet.**

We sincerely appreciate your comment. Please check the mentioned parts for more details. If there is any more comment, please inform us, and we will be very glad to make further progress.

**Reviewer#3's comment No.4:** The levels of turbulence intensities at the inlet may be specified as these may be useful for future simulations.

**Reply:** We greatly appreciate your insightful feedback. Following your feedback, we provide the levels of turbulence intensities at the inlet. Based on our observed results, the levels of turbulence intensities at the inlet are 0.0518, 0.0506, and 0.0496 which correspond to Reynolds numbers of 8,333, 10,000 and 11,666, respectively.

We sincerely appreciate your comment. Please check the mentioned parts for more details. If there is any more comment, please inform us, and we will be very glad to make further progress.

**Reviewer#3's comment No.5:** Is there only one scaling, isn't there a possibility for different scaling depending on the region?

**Reply:** We greatly appreciate your insightful feedback. Following your feedback, we provide a detail explanation. Firstly, A confined jet space is formed because of two lock walls which are defined as vertical walls in this study, based on the design manual of ship locks made by U.S. Army Corps of Engineers. The design objective of the test section is to recreate a confined 3D wall jet as accurately as feasible when water filling in lock chambers. Therefore, the width with the test section which must simulate exactly the confined space is particularly important. Most real widths of lock chambers are within the range of 12m-25m shown in table 1, resulting in the fact that 20m is selected as the prototype size to build the width of the test section in this study. Secondly, considering the experimental field, range of laser irradiation, observation range of the CCD camera, accuracy requirements for images, etc., the test section was designed according to the normal model that should obey the similarity law of gravity, and its geometric scale was determined to be 1:50 in this study. i.e., *λ_l_*=*λ_h_*=50, *λ_u_*=*λ_l_*^1/2^=7.07. Note that *λ_l_* represents the horizontal scale, *λ_h_* represents the vertical scale, and *λ_u_* represents the velocity scale, respectively. Its width was determined to be 400mm by calculation. due to difficulties in reconstructing the whole length of the lock chamber, the length of the test section was designed to be twice the width to facilitate better diffusion of a 3D wall jet in the spanwise direction. Moreover, the test section was set as 300mm high to provide greater activity space for constant-head skimming weirs. In general, the width of the test section may represent most real chamber widths, which is able to restore significantly the side orifice jet in the lock chambers when filling and ensure the correctness of the jet experiments in this study. In response to your feedback, as mentioned above, it is determined that the test section was designed on the basis of the normal model in this study, leading to the fact that the horizontal scale was equal to the vertical scale. The abnormal model was also able to be applied to design the test section. However, if it was employed, the two scales were different in the horizontal and vertical directions. The specific scales still needed to be determined according to the experimental requirements, which were the experimental field, range of laser irradiation, observation range of the CCD camera, accuracy requirements for images, etc. Most important of all, the design of the test section width must correspond to the width of lock chambers by the horizontal scale. Note that the velocity scale changed with the geometric scale.

**Table 1.** Typical ship locks with side orifices longitudinal culvert filling and emptying systems (after Zhang et al. [8])

| **Serial number** | **Names of ship locks** | **Scales of chambers**  **（Length and Width）（m）** | **Initial depths（m）** | **Designed heads（m）** | **Filling time（min）** |
| --- | --- | --- | --- | --- | --- |
| 1 | Vallee | 204×33.5 | 3.96 | 6.7 | 9.9 |
| 2 | Jackson | 204×33.5 | 3.96 | 10.86 | 10.7 |
| 3 | Colombia | 154×25 | 4.27 | 7.65 | 9.8 |
| 4 | Eisen Howen | 244×24.4 | 9.45 | 15.7 | 8.4 |
| 5 | First line of Guiping | 186×23 | 3.5 | 9.96 | 8.0 |
| 6 | First line of Dayuandu | 180×23 | 3.0 | 11.2 | 8.7 |
| 7 | First line of Guigang | 190×23 | 3.5 | 14.5 | 8.3 |
| 8 | Wheeler | 122×18.3 | 3.96 | 16.4 | 12.0 |
| 9 | Naji | 190×12 | 3.5 | 13.91 | 8.0 |

We sincerely appreciate your comment. Please check the mentioned parts for more details. If there is any more comment, please inform us, and we will be very glad to make further progress.
