# Supplementary material for: A study on turbulence characteristics of a rectangular three-dimensional wall jet in a confined space using particle image velocimetry
Source: PLoS One. 2026 May 8;21(5):e0348138. doi: 10.1371/journal.pone.0348138 (PMC13155588; doi:10.1371/journal.pone.0348138)
Supplement: Attachment — Submitted filename: Updated Response to Reviewers.docx [file pone.0348138.s005.docx]

**Authors' detailed response to the reviewers**

**(February 2026)**

**Specific reply to the Reviewers’ comments one by one**

**Reviewer#1:**

**(The specific Reviewer Number may be different between the author side and the reviewer side)**

**We sincerely appreciate your comment and recommendations, which greatly contribute to improve the quality of this paper.**

**Reviewer#1's comment No.1:** The revised manuscript has not been checked for any grammatical mistakes; the mistakes can be found at the places where they were in the first version.

**Reply:** We greatly appreciate your valuable comment. Following your feedback, we spent a long time conducting a comprehensive and systematic check for grammar errors throughout the entire paper, including grammar errors, spelling of words, tenses, etc. In addition, we have also enhanced the expression of professional vocabularies and deleted complex expressions. The professionalism and readability are promoted in the revised manuscript. Through our modifications, the revised manuscript has become more rigorous.

We sincerely appreciate your comment. Please check the revised draft for specific details. If there is any more comment, please inform us, and we will be very glad to make further progress.

**Reviewer#1's comment No.2:** If the authors believe that the self-similarity is noticed in region II (16<=x/d<24), then they should present only three results for x/d = 16, 19, and 22. This will clearly show the self-similar behaviour. In the present paper, the graph looks very clumsy. Moreover, the authors are suggested to use the same symbols in all the figures pertaining to a particular location. Please modify the figures accordingly.

**Reply:** We greatly appreciate your valuable comment. Following your feedback, we give a detailed explanation.

(i) In our previous studies (Refs. [20] and [21]), the self-similarity of mean statistics was shown in the region 16≤*x/d*<24 (region Ⅱ). However, turbulence statistics in region Ⅱ present the different rule. To explore it, the typical profiles of turbulence statistics are shown in Fig.8, including the profiles at 16d,17d, 18d, 19d, 20d, 21d, 22d, 23d. In general, the measured values of *u*, *v*, and *uv* have an increasing trend along the streamwise direction in the region 16≤x/d<19 both in the symmetry plane and lateral plane. The growth trend is disappeared, and profiles of *u*, *v*, and *uv* show reasonable collapse in the region 19≤x/d<24 (5*d*). This illustrates that turbulence statistics in region Ⅱ require a relatively longer streamwise distance (3*d*) to become self-similar than mean characteristics. This behavior is physically expected: turbulence kinetic energy of fluctuating motions is generated by mean motions; thus, the self-similarity of the mean flow development must be self-similar before the turbulence quantities. Therefore, we need to present the turbulence statistics profiles in the region 16≤*x/d*<24 to observe the changes and draw the above conclusion through our experiments.

(ii) The drawing and representation methods of the graphs in the present paper are consistent with the previous studies on the three-dimensional wall jet, including the confined case and unconfined case. Displaying and analyzing the statistical distribution of each profile in the specific flow region is an effective way to study the flow mechanism of a three-dimensional wall jet. In each figure, we have clearly marked the legend to help the readers understand the experimental profiles data. In addition, we have also provided detailed explanations for each figure titles. This can promote the readability of the graphs in this paper. In summary, if different symbols appear in the figures, it will not cause comprehension difficulties for readers.

We sincerely appreciate your comment. If there is any more comment, please inform us, and we will be very glad to make further progress.

**Reviewer#2:**

**(The specific Reviewer Number may be different between the author side and the reviewer side)**

**We sincerely appreciate your comment and recommendations, which greatly contribute to improve the quality of this paper.**

**Reviewer#2's comment:** The authors have adequately addressed the questions and concerns raised in the previous review. Their responses were clear, comprehensive, and supported by appropriate revisions to the manuscript. Based on the improvements made and the satisfactory resolution of the issues, I recommend the manuscript be accepted for publication.

**Reply:** We greatly appreciate your insightful feedback. Thanks.

**Reviewer#3:**

**(The specific Reviewer Number may be different between the author side and the reviewer side)**

**We sincerely appreciate your comment and recommendations, which greatly contribute to improve the quality of this paper.**

**Reviewer#3's comment:** The authors have adequately addressed the concerns raised by the present reviewer. Therefore, I recommend the publication of this manuscript. Thank you.

**Reply:** We greatly appreciate your insightful feedback. Thanks.
